# Kinases of the Focal Adhesion Complex Contribute to Cardiomyocyte Specification

**DOI:** 10.3390/ijms221910430

**Published:** 2021-09-28

**Authors:** Sacha Robert, Marcus Flowers, Brenda M. Ogle

**Affiliations:** 1 Department of Biomedical Engineering, University of Minnesota-Twin Cities, Minneapolis, MN 55455, USA; srobert@umn.edu (S.R.); flowe105@umn.edu (M.F.); 2 Stem Cell Institute, University of Minnesota-Twin Cities, Minneapolis, MN 55455, USA; 3 Lillehei Heart Institute, University of Minnesota-Twin Cities, Minneapolis, MN 55455, USA; 4 Institute for Engineering in Medicine, University of Minnesota-Twin Cities, Minneapolis, MN 55455, USA; 5 Masonic Cancer Center, University of Minnesota-Twin Cities, Minneapolis, MN 55455, USA

**Keywords:** biomaterials, stem cells, signalization, focal adhesion kinases, heart, scaffolds, tissue engineering, cardiomyogenesis, extracellular matrix

## Abstract

Differentiation of pluripotent stem cells to cardiomyocytes is influenced by culture conditions including the extracellular matrices or similar synthetic scaffolds on which they are grown. However, the molecular mechanisms that link the scaffold with differentiation outcomes are not fully known. Here, we determined by immunofluorescence staining and mass spectrometry approaches that extracellular matrix (ECM) engagement by mouse pluripotent stem cells activates critical components of canonical wingless/integrated (Wnt) signaling pathways via kinases of the focal adhesion to drive cardiomyogenesis. These kinases were found to be differentially activated depending on type of ECM engaged. These outcomes begin to explain how varied ECM composition of in vivo tissues with development and in vitro model systems gives rise to different mature cell types, having broad practical applicability for the design of engineered tissues.

## 1. Introduction

Regenerative therapies for cardiac tissue are in high demand to treat a number of conditions including congenital heart defects, ischemic injury, and myocarditis [1,2]. Combined, these maladies represent more than a third of annual deaths in the United States. 3D bioprinting structures that mimic the composition of native tissue have been proposed as a therapeutic option, gaining traction due to their accessibility and modularity [3,4,5]. While this is an exciting area of research, it comes with substantial challenges, particularly with cardiac tissue. Inclusion of mature cardiomyocytes is challenging as they cannot migrate to populate the 3D printed tissue mass with proper spatial organization and are further a non-proliferative cell type. On the other hand, in situ differentiation of stem cells or their progeny requires cues for differentiation, and these cues differ between the cell types found in native heart tissue.

We reason that tailored cues from the extracellular matrix (ECM) may be used to overcome the challenge of differentiating multiple cell types in an engineered tissue with spatial acuity. Decades-old prior works have demonstrated that production and engagement of ECM often precedes differentiation events. More recently, a growing number of studies show the same is true in ex vivo systems containing stem cells [6,7]. In our work, we show that the ECM, independent of exogenous growth factor or small molecule stimulation, can effectively drive cardiomyocyte differentiation [2,8,9].

The next important step is to identify the intracellular mechanisms that link ECM engagement with stem cell specification. Current literature has established that mesoderm specification is linked to α5β1 integrin activation. Engagement of this integrin by ECM (especially laminin-511/111 and fibronectin) modulates BMP4 expression, which together with Wnt, fibroblast growth factor and transforming growth factor-β/nodal/activin signaling, mediates differentiation [10]. Further, it has been shown that fibronectin/integrin β-catenin signaling can promote the emergence of mesoderm from induced pluripotent stem cells. Cheng et al. was the first to establish a direct link between elements of the focal adhesion, namely, integrin-linked kinase (ILK), with glycogen synthase kinase 3 beta (GSK3β) [11], the primary antagonist of β-catenin.

In this study, we build on our understanding of ECM engagement and stem cell differentiation by discerning the pathways linking focal adhesion (stem and progenitor cells) or costamere (cardiomyocyte) formation with mesoderm specification and cardiomyocyte differentiation with ECM substrates that optimally or suboptimally support cardiomyogenesis in the absence of exogenously provided growth factor and small molecule signals. We anticipate this knowledge will help develop strategies to either maintain cell potency in ECM-based matrices or promote cell differentiation in ECM-based matrices. We focused on three candidate kinases, namely, ILK, the serine/threonine p21-activated kinase (PAK), and the focal adhesion kinase (FAK), which have been proposed as important modulators of ECM-associated signaling pathways. Our findings demonstrate early dynamical changes in the ECM composition during cardiomyocyte differentiation concomitant with mesoderm expression, which are mainly regulated by FAK signaling in 2D culture of mouse induced pluripotent stem cells (miPSCs).

## 2. Results

### 2.1. Engagement of Optimized ECM Formulation with miPSCs in 2D Induced Cardiomyocyte Differentiation

**Figure 1:**
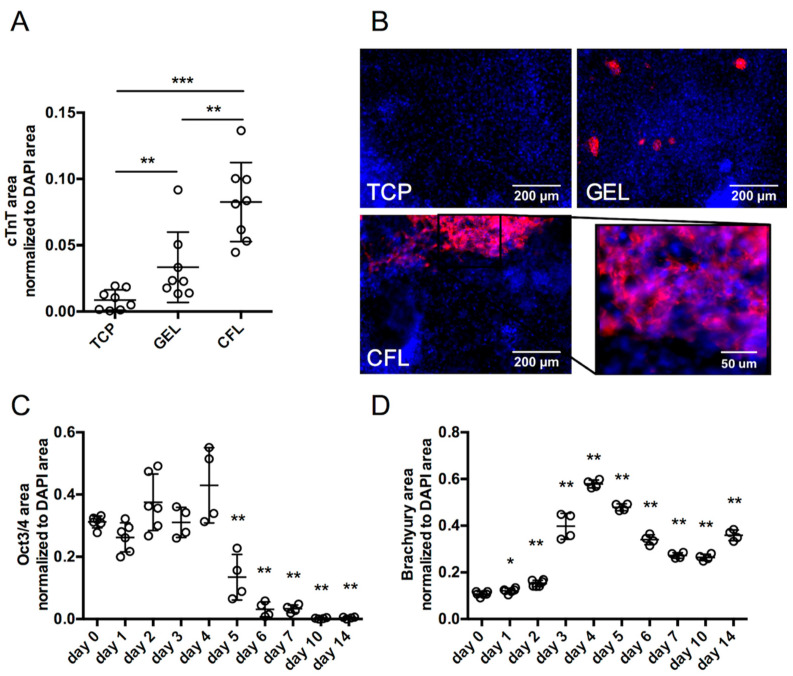
Differentiation of miPSCs into cardiomyocytes upon ECM engagement. (**A**,**B**) Immunofluorescence staining of cTnT at 14 days of culture with tissue culture plastic (TCP), gelatin 0.1 % (GEL), or ECM mixture comprised of collagen type I, fibronectin, and laminin-111 (CFL) and its associated quantification of cTnT staining normalized to DAPI area. (**C**) Quantification of pluripotency marker Oct3/4 immunofluescence staining at different time points of differentiation of miPSCs upon CFL culture condition. (**D**) Quantification of mesodermal marker Brachyury/T immunofluorescence staining at different time points of differentiation of miPSCs upon CFL culture condition. Comparison of mean between TCP, GEL, and CFL or day 0 condition to the other days. * *p* < 0.05, ** *p* < 0.01, *** *p* < 0.001, unpaired *t*-test with Mann–Whitney *U* test.

We have previously identified an ECM formulation capable of supporting cardiomyocyte differentiation in 3D hydrogels without the addition of soluble factors [2]. In order to more accurately identify the signaling pathways transduced by ECM leading to cardiac differentiation of miPSCs, we adapted the formulation consisting of type I collagen, fibronectin, and laminin-111 (CFL) for a 2D cell culture system. The 2D system allows for accurate imaging with immunofluorescence and for easier isolation of cells for protein quantification without the possibility of modulating cell signaling during harsh enzymatic degradation of 3D matrices. The capacity for cardiomyocyte differentiation of miPSCs was evaluated in several 2D coating conditions including a no coating negative control (tissue culture plastic, TCP), a gelatin coating (GEL) as an ECM protein known to support miPSC adhesion but not cardiomyocyte differentiation, and CFL. To determine whether CFL preferentially promotes cardiomyocyte differentiation in the absence of other exogenous signals, we seeded miPSCs on each of TCP, GEL, and CFL. Fourteen days after seeding, cardiac troponin T (cTnT) expression was measured via immunofluorescence staining for each condition as an indicator of cardiomyocyte differentiation. Both the GEL and the CFL substrates supported a significantly higher level of cardiomyocytes compared to the control (TCP). Moreover, CFL is more potent to induce cardiomyocytes than GEL (Figure 1A). This increased cardiomyocyte marker is accompanied by spontaneous beating areas of cells that could be observed in the CFL condition as soon as day 8 of differentiation (Video S1), reflecting another cardiomyocyte feature. We further probed dynamic changes in the expression of the pluripotency marker Oct3/4 and the precardiac mesoderm marker brachyury (T) following exposure of miPSCs to CFL. Indeed, CFL coating induces a significant, time-dependent decrease in Oct3/4 in the early days of culture leading to it complete loss by day 14 (Figure 1C). In parallel, brachyury expression is induced in the first day of culture to reach its maximum by day 4 before starting to decrease and plateau at a level that is maintained until day 14 (Figure 1D).

### 2.2. FAK and ILK Signaling Pathways Regulated Cardiomyocyte Differentiation of miPSCs upon ECM Engagement

**Figure 2:**
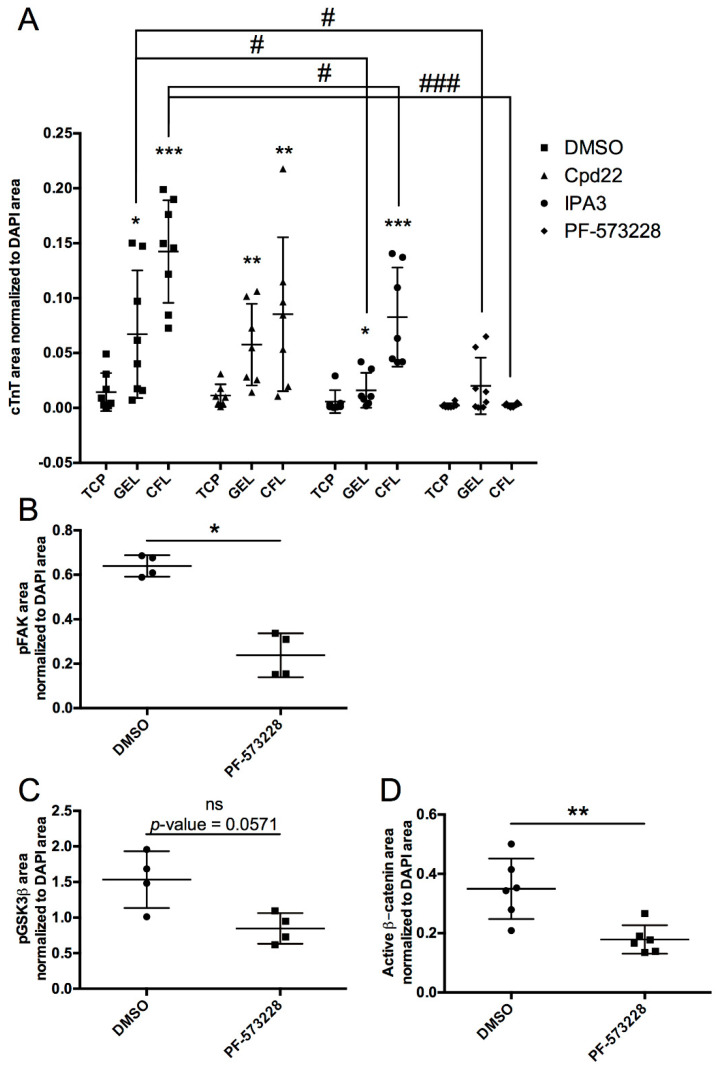
Differentiation of miPSCs into cardiomyocytes upon ECM engagement is secondarily associated with ILK/PAK-1 signaling and primarily associated with FAK signaling, potentially linked to Wnt signaling. (**A**) cTnT immunofluorescence staining of miPSCs after 14 days of culture in TCP, GEL, or CFL conditions with daily treatment with the control vehicle (0.1 % DMSO); ILK-specific inhibitor; Cpd22 at 0.4 µM; PAK-1-specific inhibitor; IPA3 at 1 µM; or FAK-specific inhibitor, PF-573228 at 1 µM. Comparison of mean against TCP for each group. * *p* < 0.05, ** *p* < 0.01, *** *p* < 0.001 unpaired *t*-test with Mann–Whitney *U* test. Comparison of means between conditions within each group. # *p* < 0.05, ### *p* < 0.001, unpaired *t*-test with Mann–Whitney *U* test. (**B**) Phosphorylated (Y397)-FAK immunofluorescence staining area normalized to DAPI area of TCP, GEL, and CFL conditions after 4 days of culture with PF-573228 (1 µM). (**C**) Phosphorylated (S9)-GSK3β immunofluorescence staining area normalized to DAPI area of TCP, GEL, and CFL conditions after 4 days of culture with PF-573228 (1 µM). (**D**) Active β-catenin immunofluorescence staining area normalized to DAPI area of TCP, GEL, and CFL conditions after 4 days of culture with PF-573228 (1 µM). Comparison of means between vehicle and inhibitor. * *p* < 0.05, ** *p* < 0.01, unpaired *t*-test with Mann–Whitney *U* test.

Among several cellular signaling molecules activated by ECM engagement, we sought to investigate the role of kinases ILK, PAK-1, and FAK in cardiomyocyte differentiation of miPSCs. These kinases were selected because they have been shown to be directly or indirectly linked to the Wnt signaling pathway via regulation of GSK3β, a critical node for cardiomyogenesis [11,12,13]. We employed Cpd22, IPA3, and PF-573228, specific chemical inhibitors of ILK, PAK-1, or FAK activation, respectively. As shown in Figure 2A, while all the inhibitors decreased CFL-induced miPSCs cardiomyocyte differentiation, only IPA3 and PF-573228 effects were statistically significant. Similarly, when miPSCs were cultured with gelatin coating, only PAK-1 and FAK inhibitors led to a significant decrease of miPSCs cardiomyocyte differentiation (Figure 2A). These data suggest that differentiation of miPSCs into cardiomyocytes upon ECM engagement involves unique combination of ECM-activated kinases depending on the ECM engaged. Of the three inhibitors, Cpd22 and IPA3 showed only partial knockdown, suggesting compensatory pathways are possible. In contrast, PF-573228, completely abrogated cardiomyocyte differentiation of miPSCs upon ECM engagement, suggesting an essential role of FAK in driving ECM-mediated cardiomyocyte specification. In further support, FAK inhibition resulted in a significant reduction in p-(Y397) FAK levels (Figure 2B), which was associated with a decreased in p-(S9) GSK3β and a significant decrease of the active form of β-catenin levels at day 4 of differentiation (Figure 2C). We also found that, while total FAK levels were unaffected by coating conditions, p-FAK level was significantly increased in GEL and CFL conditions compared to the control TCP at day 4 of differentiation (Supplementary Figure S1). Overall, these data suggest that the activation of FAK upon ECM engagement leads to the activation of GSK3β, which could activate canonical Wnt/β-catenin pathway, a key event in mesoderm and cardiomyocyte fate of miPSCs [12].

### 2.3. ECM-Engagement with miPSC Included Characteristic Changes in ECM Composition during Cardiomyocyte Differentiation

**Figure 3:**
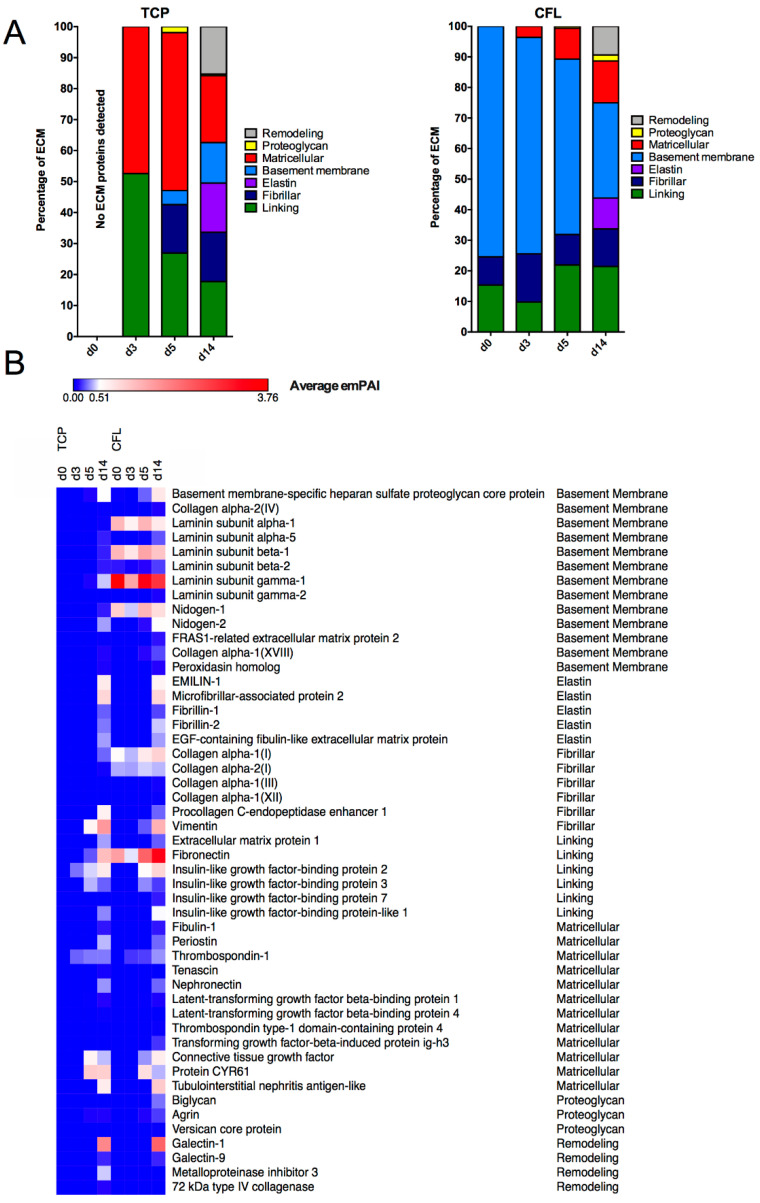
Dynamic changes of ECM proteins composition in miPSC cultures following ECM-driven cardiomyocyte differentiation. (**A**) Proteomic analysis of ECM families in TCP or CFL cultures at days 0, 3, 5, and 14 that was based on the emPAI number, determined with LC–MS^2^. (**B**) Heatmap of all identified ECM proteins based on the average emPAI number to estimate the level of proteins in samples.

The key role of the canonical Wnt/β-catenin pathway activation in mesoderm specification of miPSCs is well described in the literature [14]. However, in order for cardiomyocyte differentiation to be achieved, the downregulation of Wnt/β-catenin pathway after mesoderm specification is required [12]. We hypothesized that CFL exposure induces the activation of Wnt/β-catenin to initiate mesoderm formation and that downregulation of Wnt/β-catenin is achieved via ECM remodeling. Employing a mass spectrometry-based proteomics approach, we analyzed temporal changes in the composition of ECM proteins during cardiomyocyte differentiation of miPSCs (Figure 3A). As expected, at day 0, no ECM proteins were detected in TCP, while type I collagen, laminin-111, and fibronectin (classified as fibrillar for collagen, basement membrane for laminin, and linking for fibronectin) were detected in CFL. After 3 days, corresponding to pre-mesoderm induction, matricellular and linking family proteins were detected in TCP condition, and matricellular proteins in CFL condition, suggesting the production and deposition of ECM by miPSCs. By day 3 in the CFL condition, the matricellular family started to rise. At day 5, corresponding to cardiomyocyte precursor specification, in TCP condition, the ECM composition further diversified by the inclusion of proteoglycan, basement membrane, and fibrillar family proteins detected in addition to the matricellular and linking family. In the CFL condition, however, no further ECM family changes were detected at day 5 in comparison to day 3. Interestingly, by day 14, both TCP and CFL conditions showed similar relative protein family composition, with the appearance of remodeling and elastin ECM proteins for both conditions, and proteoglycan family in CFL condition. Considering the differences in cardiomyogenesis between TCP and CFL, we found that these results suggest ECM remodeling may drive specification to the germ layer designation, and further to their derivatives.

To more specifically delineate candidate ECM proteins involved in cardiomyocyte specification, we employed a heatmap of individual ECM proteins over time. The heatmap was generated on the basis of average emPAI values, and single ECM proteins were organized by ECM families (Figure 3B). Apart from the initial ECM proteins from the CFL condition (i.e., type I collagen, laminin-111, and fibronectin), we identified several other differentially expressed ECM proteins between TCP and CFL conditions at days 3 and 5 of differentiation. Basement membrane-specific heparan sulfate proteoglycan core protein, nidogen-2, type XVIII collagen (alpha-1), transforming growth factor-beta-induced protein ig-h3, and biglycan are either absent or expressed only by day 14 in the TCP condition, compared to the CFL condition. In contrast, insulin-like growth factor-binding protein 2, metalloproteinase inhibitor 3, and 72 kDa type IV collagenase are either absent or expressed only by day 14 in the CFL condition, compared to the TCP condition. Altogether, these data support the possibility that ECM remodeling contributes to mature cell differentiation following ECM-mediated mesoderm specification.

## 3. Discussion

Extracellular matrix proteins have a strong influence on stem cell differentiation. The specification of more than 20 cell types has been linked to engagement of one or a cadre of ECM proteins [15]. Here, we characterized the major signaling pathways involved in cardiomyocyte differentiation of miPSCs upon ECM engagement. We found that ECM engagement linked to activation of kinases of the focal adhesion complex was critical for cardiomyocyte specification and further that kinase activity was associated with Wnt/β-catenin signaling.

The canonical Wnt pathway is a prominent cell signaling pathway that plays a key role in directing cardiomyocyte differentiation [16]. Wnt signaling is activated through inhibition of either Wnt or GSK3β, both of which result in an increase in β-catenin within the nucleus [17]. It is well established that GSK3β inhibition is catalyzed by several kinases, among them being ILK and FAK, which had been related to ECM signaling [11,13]. Thus, we hypothesized that ECM engagement could modulate ILK or FAK activity, leading to Wnt pathway activation associated with cardiomyocyte differentiation. PAK-1, a cytoskeletal-associated kinase activated by small GTP binding proteins, is an ILK and FAK downstream signaling intermediate [18,19] that could fill the gap between integrin-dependent kinases activation and Wnt signaling. Our hypothesis is supported by several studies showing the involvement of type I collagen, fibronectin, or laminin-111 in ILK and FAK activation [20,21]. Employing PF-573228 and Cpd22, specific inhibitors of FAK and ILK, respectively, we observed a decrease in cardiomyocyte differentiation of miPSCs. However, compared to ILK, FAK inhibition exhibited the greatest reduction of cardiomyocyte differentiation. While the activation of ILK and FAK are clearly involved in cardiomyocyte differentiation, their association with Wnt activation remain contested. ILK has been shown to induce the phosphorylation of GSK3β on Ser-9 [22] and to co-immunoprecipitate with phospho-GSK3β, an event mediating the emergence of mesoderm from mESCs upon the fibronectin/integrin β1/β-catenin signaling axis [11]. While it has never been reported that activated FAK could act directly on GSK3β as a substrate for phosphorylation on Ser-9 like ILK can, we found that PF-573228 was able to downregulate p-(S9)-GSK3β and the active form of β-catenin. Thus, activated FAK can induce GSK3β phosphorylation in miPSCs, leading to cardiomyogenesis regulation through the canonical Wnt/β-catenin signaling pathway upon ECM engagement. Interestingly, it has been reported that FAK can directly phosphorylate GSK3β on Tyr-216, a mechanism necessary for activation of Wnt/β-catenin signaling [13]. Similarly, we found that PAK-1 inhibition also resulted in decreased cardiomyocyte differentiation. PAK-1 has been shown to directly phosphorylate β-catenin on Ser-675 residue, leading to a more stable and transcriptionally active form of β-catenin [23], reinforcing the prominent role of Wnt signaling in the control of cardiac differentiation upon ECM engagement.

The ECM–integrin–kinase–Wnt pathway supports Wnt activation and therefore the onset of mesoderm specification. However, this alone does not explain which signals drive cardiomyocyte differentiation. One possibility is that ECM engagement at early stages of specification drives new ECM production to enable later stages of differentiation. To test this possibility, we employed semi-quantitative mass spectrometry to track ECM production and accumulation with and without ECM-guided differentiation. Our data demonstrated the early production and the persistence of collagen XVIII, a marker of immature cardiomyocytes associated with fetal myocardium that is also secreted by immature cardiomyocytes [24]. However, the role of collagen XVIII remains unclear. We also demonstrate the production of basement membrane-specific heparan sulfate proteoglycan core protein (e.g., perlecan) throughout cardiomyocytes differentiation, which reportedly plays an important role in maintaining tissue integrity during cardiac development and post-injury adult hearts [25]. Interestingly, we find nidogen-2 production in the CFL condition; nidogens act as connecting elements between the collagen IV and laminin networks to integrate other basement membrane components, including perlecan [26].

Alongside perlecan, we also identify biglycan as another interesting proteoglycan. Biglycan has been shown to induce hypertrophy of cardiomyocytes, suggesting that biglycan may act as a signaling molecule between cell types to modulate cardiac remodeling [27]. Hence, our data suggest an important role of the interplay between basement membrane and proteoglycan proteins for the growth of cardiomyocyte precursor cells. Additional studies are required to better understand the role of these proteins in the maturation of miPSC-derived cardiomyocytes.

We report the expression of remodeling ECM proteins, TIMP3, and 72kDa type IV collagenase in the TCP condition and not the CFL condition. The major functionality of TIMP-3 is matrix remodeling through the regulation of disintegrin and metalloproteinases (ADAMs) [28]. The 72kDa type IV collagenase is known to contribute to myocardial oxidative stress by regulating the activity of GSK3β [29] but can also help forming dense vascular network close to the native myocardium when stem cells are co-cultured with endothelial cells [30]. Thus, future work will test whether TIMP-3 and type IV collagenase could remodel the ECM in our 2D cell culture system needed for cardiomyocyte differentiation.

**Scheme 1:**
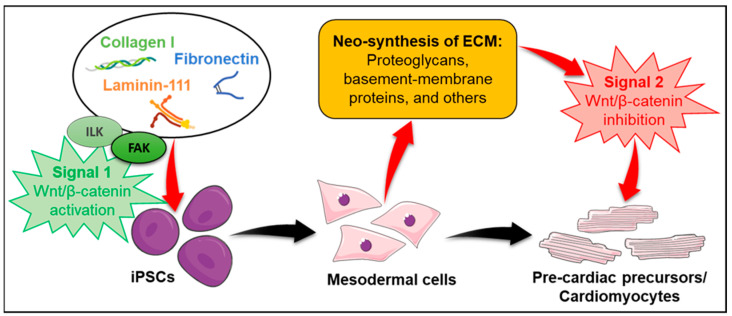
Proposed mechanism underlying miPSC differentiation into cardiomyocytes upon dynamical ECM engagement.

Overall, our study shows the validity of the 2D cell culture system to support cardiomyocyte differentiation of miPSCs. Using this accessible cell culture system, we were able to identify for the first-time dynamic changes in ECM proteins of miPSCs throughout ECM-induced cardiac differentiation, via a semi-quantitative mass spectrometry-based approach. On the molecular level, we were able to highlight FAK as a major player in cardiac differentiation and establish a link between ECM engagement with integrins and Wnt/β-catenin signaling (Scheme 1). In the future, we envision that a tight regulation of FAK signaling in conjunction with soluble factors can be used to enhance the functional capacity of cardiomyocytes derived from PSCs. Future studies should focus on unravelling the roles of the dynamical changes in ECM protein expression, such as collagen XVIII, perlecan, biglycan, 72kDa type IV collagenase, and TIMP-3 during cardiac differentiation. A temporal comparison between our mass spectrometry data and RNA sequencing could help to further identified important molecules for that matter. Potential improvements can be made by verifying the functional interaction of FAK with GSK3β, both in 2D and 3D systems, and determining whether this signaling pathway is conserved with human cells. Investigating which integrins are required in this process can also help future studies in the choice of ECM substrates for differentiation of cardiac cells and cells of other tissue types.

## 4. Materials and Methods

### 4.1. Cell Culture

miPSC were obtained from Deepak Srivastava at the Gladstone Institute of Cardiovascular Disease (Mutant Mouse Resource and Research Centers 030440, mixed strain 129OLA/C57BL/6). miPSC purification was performed as previously described [31]. For maintenance or pluripotency, miPSCs were cultured on gelatin-coated (Sigma-Aldrich, St. Louis, MO, USA), 6-well plates at a density of 3700 cells/cm^2^ in modified GMEM (Sigma-Aldrich, St. Louis, MO, USA) supplemented with 10 % fetal bovine serum (FBS) (Gibco, Life Technologies, Carlsbad, CA, USA), nonessential amino acids (Gibco, Life Technologies, Carlsbad, CA, USA), L-glutamine (2 mM) (Gibco, Life Technologies, Carlsbad, CA, USA), β-mercaptoethanol (0.1 mM) (MP Biomedical, Solon, OH, USA), and 2000 units/mL leukemia inhibitory factor (MilliporeSigma, Burlington, MA, USA). Cells were subcultured using 0.05% Trypsin/EDTA (Gibco, Life Technologies, Carlsbad, CA, USA) when 60–70% confluent.

### 4.2. Cardiomyocyte Differentiation upon ECM Engagement

One day prior to miPSCs seeding (day 1), CFL-condition 96-well plates were coated with a mixture of 15 µg fibronectin (Cat# 356008, Corning Inc., Corning, NY, USA), 24 µg laminin/entactin (Cat# 354259, Corning Inc., Corning, NY, USA), and 61 µg collagen type I (Cat# 354249, Corning Inc., Corning, NY, USA), all mixed in deionized ultra-filtered water and incubated at 4 °C overnight. The next day (day 0), the plates were washed twice with sterile phosphate-buffered saline (PBS) prior to miPSC seeding. The gelatin condition was coated with 0.1 % gelatin for 5 min at room temperature before miPSCs seeding. miPSCs were plated in modified GMEM without LIF at 7000 cells/cm^2^. The next day (day 1), media was replaced with unmodified GMEM (Cat# G5154, Sigma-Aldrich, St. Louis, MO, USA) and changed every day for 2 weeks, with or without the FAK inhibitor, PF-573228 at 1 µM in DMSO (Cat# 3239, Tocris Bioscience, Bristol, UK); the ILK inhibitor, Cpd22 at 0.4 µM in DMSO (Cat# 407331, MilliporeSigma, Burlington, MA, USA); the PAK-1 inhibitor, IPA-3 at 1 µM in DMSO (Cat# 3622, Tocris Bioscience, Bristol, United Kingdom); or 0.1 % DMSO as vehicle control.

### 4.3. Decellularization

At specified time points after ECM-guided differentiation, cells were removed from the surrounding matrix. First, the media was aspirated, and cells were rinsed twice with PBS. Then, cells were washed three times with wash buffer 1 (100 mM Na_2_HPO_4_, 2 mM MgCl_2_, 2 mM EDTA; pH 9.6), followed by the addition of lysis buffer (8 mM Na_2_HPO_4_, 1 % Triton X-100; pH 9.6), and incubated for 15 min at 37 °C. The lysis buffer was then replaced with fresh lysis buffer and incubated for 40 min at 37 °C. The lysis buffer was removed and rinsed three times with wash buffer 2 (10 mM Na_2_HPO_4_, 300 mM KCl; pH 7.5), followed by four washes with DI water. After the final wash, PBS was added to the wells and stored at 4 °C before trypsin digestion.

### 4.4. Mass-Spectrometry Analysis

Decellularized samples were homogenized in lysis buffer containing 6 M urea and 100 mM tris (pH 7.8), and pipetted down for 2 min. Samples were then reduced using 200 mM DTT in Tris buffer and incubated for one hour at room temperature. Next, samples were alkylated using 200 mM iodoacetamide in tris buffer and incubated for one hour at room temperature in the dark. DTT reducing agent was added once more to consume any unreacted iodoacetamide and incubated for another hour at room temperature in the dark. Urea concentration was then reduced to 1 M by diluting the reaction mixture with 1 mM CaCl_2_ solution. The pH was checked and adjusted between 7.8 and 8.7 to allow trypsin (#V5280, Promega, Madison, WI, USA) activity at 2 µg per well, and the mixture was incubated overnight at 37 °C. The following day, the reaction was stopped by lowering the pH to 3.5 with formic acid. Peptides were then collected into Eppendorf tubes and desalted and filtered through a C18 Sep-Pak cartridge (#WAT020215, Waters, Milford, MA, USA); then, they were eluted from the C18 bed using 65 % acetonitrile/0.1 % formic acid. The organic component was removed by evaporation in a vacuum concentrator (Savant SpeedVac, Thermo Fisher Scientific, Waltham, MA, USA), and peptides were resuspended in 20 µL of 2 % acetonitrile/0.1 % formic acid and analyzed by LC–MS on the Orbitrap Elite mass spectrometer (Thermo Fisher Scientific, Waltham, MA, USA). Raw data were processed with ProteomeDiscoverer software (Thermo Fisher Scientific, Waltham, MA, USA) and UniProt (www.uniprot.org, accessed on 31 May 2018) database using the tryptic digestion rule.

Identified proteins were sorted into 7 groups on the basis of their ECM characteristics and functions, and average exponentially modified protein abundance indices (emPAI) were calculated and reported as portions of total ECM and total protein.

### 4.5. Immunofluorescence Staining

Primary antibodies used included anti-cTnT (mouse IgG, clone 13-11, Cat# MS-295-P1, 1:200, Thermo Fisher Scientific, Waltham, MA, USA), p-(Y397), anti-pFAK (rabbit IgG, clone 31H5L17, Cat# 700255, 1:200, Thermo Fisher Scientific, Waltham, MA, USA), anti-brachyury (goat IgG, Cat# AF2085, 1:50, R&D systems, Minneapolis, MN, USA), anti-Oct3/4 (mouse IgG2b, clone C-10, Cat# sc-5279, 1:200, Santa Cruz Biotechnology, Dallas, TX, USA), anti-p-S9 GSK3β (rabbit IgG, clone 5B3, Cat# 9323, 1:100, Cell Signaling Technology, Danvers, MA, USA), anti-total FAK (mouse IgG2b, clone 34Q36, Cat# AHO1272, 1:200, Thermo Fisher Scientific, Waltham, MA, USA), and non-phosphorylated (active form) β-catenin (rabbit IgG, clone D13A1, Cat# 8814S, 1:200, Cell Signaling Technology, Danvers, MA, USA), and secondary antibodies were goat anti-mouse IgG Alexa Fluor 647 (Cat# A21236, 1:500, Thermo Fisher Scientific, Waltham, MA, USA), goat anti-rabbit IgG Alexa Fluor 647 (Cat# A32733, 1:500, Thermo Fisher Scientific, Waltham, MA, USA), and donkey anti-goat IgG Alexa Fluor 488 (Cat# 32814, 1:500, Thermo Fisher Scientific, Waltham, MA, USA). Immunofluorescence staining was conducted as reported previously [4]. Thirty-five fields of view per well, two wells per replicate, and two replicates per condition were taken using a 10× magnification lens on a Leica DMi8 fluorescence microscope (Leica Camera, Wetzlar, Germany). These were combined as tilescans and quantified for positive fluorescence relative to negative controls and then normalized to the DAPI (nucleus) fluorescence for the same fields of view. According to our estimation, each field of view contained ≈2750 cells. Our analysis of the percentage of DAPI area (nuclei) and average number of nuclei per a given field of view demonstrated that miPSCs maintained consistent cell density when grown on various ECM substrates and with different small molecule treatments (Supplementary Figure S2), validating the quantification method normalized to the DAPI area. Quantification was achieved using ImageJ, converting images to 8-bit, adjusting brightness/contrast to remove signals detected in secondary antibody negative controls, and thresholding before measuring.

### 4.6. Statistical Analysis

Data were expressed as mean ± standard deviation (SD). Student’s unpaired *t*-test were used to determine statistical significance between groups when the distribution of the samples in a given condition was normal. Normal distribution of samples was evaluated by two normality tests (D’Agostino and Pearson omnibus, and Shapiro–Wilk). For groups that did not pass the normality test, non-parametric statistical comparisons were conducted using the Mann–Whitney *U* test. A value of *p* < 0.05 was considered statistically significant (* *p* < 0.05; ** *p* < 0.01, *** *p* < 0.005 versus control).

## Data Availability

The raw data supporting the conclusions of this article will be made available by the authors, without undue reservation, to any qualified researcher.

## Supplementary Materials

The following are available online at https://www.mdpi.com/article/10.3390/ijms221910430/s1.

## Abbreviations

BMP4	bone morphogenetic protein 4
CFL	collagen type I, fibronectin, laminin-111
cTnT	cardiac troponin T
DI	deionized
DTT	dithiothreitol
ECM	extracellular matrix
EDTA	ethylenediaminetetraacetic acid
emPAI	exponentially modified protein abundance index
FAK	focal adhesion kinase
FBS	fetal bovine serum
GEL	gelatin
GMEM	Glasgow minimum essential medium
GSK3β	glycogen synthase kinase-3 beta
ILK	integrin-linked kinase
LC-MS^2^	liquid chromatography with tandem mass spectrometry
LIF	leukemia inhibitory factor
miPSC	mouse induced pluripotent stem cell
PAK	p21 activated kinase
PBS	phosphate-buffered saline
SD	standard deviation
T	brachyury
TCP	tissue culture plastic
