# Supplementary material for: Kinases of the Focal Adhesion Complex Contribute to Cardiomyocyte Specification
Source: Int J Mol Sci. 2021 Sep 28;22(19):10430. doi: 10.3390/ijms221910430 (PMC8508671; doi:10.3390/ijms221910430)
Supplement: Supplementary file 1 [file ijms-22-10430-s001.zip › ijms-1349989-supplementary.pdf]

Kinases of the focal adhesion complex contribute to cardiomyocyte specification

Sacha Robert [1,2], Marcus Flowers [1,2] and Brenda M. Ogle [1-5*]

Supplementary Information

[1] Department of Biomedical Engineering, University of Minnesota – Twin Cities, Minneapolis, MN 55455 USA
[2] Stem Cell Institute, University of Minnesota – Twin Cities, Minneapolis, MN 55455 USA
[3] Lillehei Heart Institute, University of Minnesota – Twin Cities, Minneapolis, MN 55455 USA
[4] Institute for Engineering in Medicine, University of Minnesota – Twin Cities, Minneapolis, MN 55455 USA
[5] Masonic Cancer Center, University of Minnesota – Twin Cities, Minneapolis, MN 55455 USA

* Correspondence: Author: Brenda M. Ogle, PhD, Department of Biomedical Engineering, University of Minnesota – Twin Cities, 312 Church St. SE, Minneapolis, MN 55455. Phone: 612-624-5948. Fax: 612-626-6583. E-mail: ogle@umn.edu

Video S1: miPSC-derived cardiomyocytes generated on the CFL substrate were capable of spontaneous beating. miPSCs were seeded onto CFL-coated plates and incubated for 8 days. Bright field images were captured using an Axiovert microscope (Zeiss) into the 10x magnification and images were stacked into ImageJ to create a 7 frames per second video.

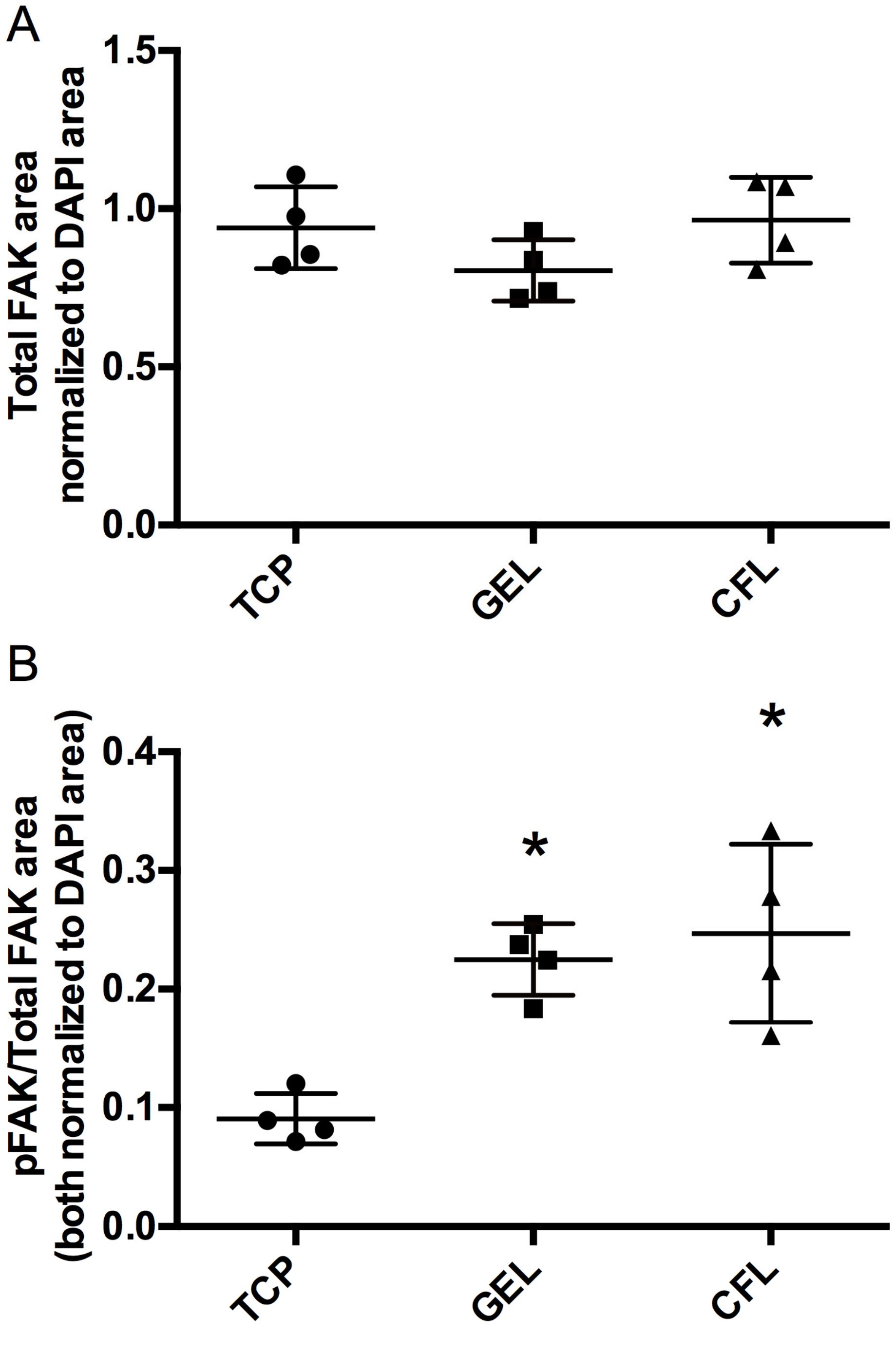

Figure S1: Differentiation of miPSCs into cardiomyocytes upon ECM engagement does not affect total FAK levels but increased p-FAK levels. (**A**) Non-phosphorylated (total)-FAK immunofluorescence staining area normalized to DAPI area of TCP, GEL and CFL conditions after 4 days of culture. (**B**) Phosphorylated (Y397)-FAK immunofluorescence staining area normalized to total FAK area (after DAPI area normalization for both) of TCP, GEL and CFL conditions after 4 days of culture. Comparison of means to the TCP condition. *P<0.05, unpaired t test with Mann-Whitney U test (n=4).

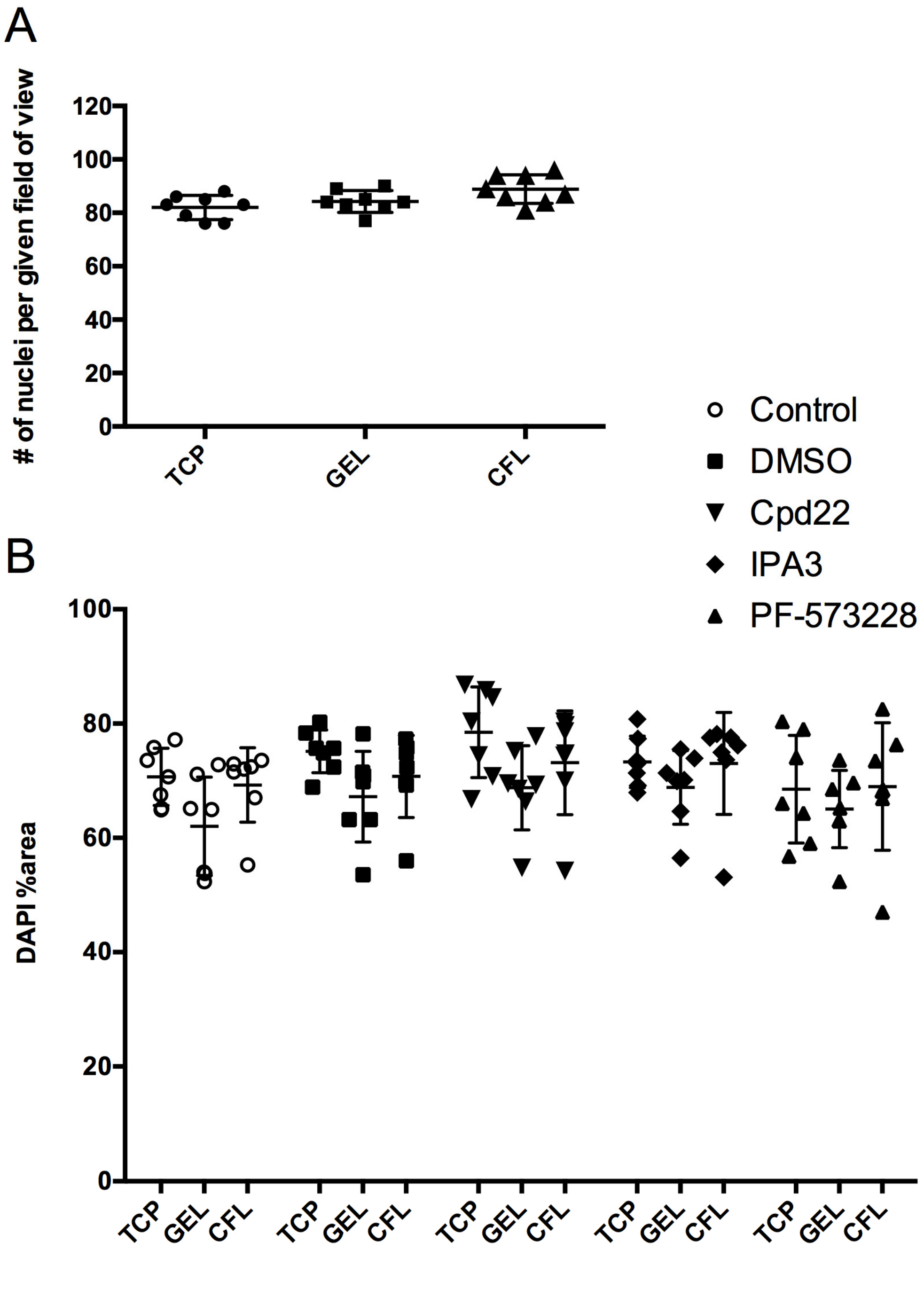

Figure S2: ECM engagement or chemical inhibition of focal adhesion signaling pathways does not affect cell density. (**A**) The number of nuclei in a given field of view were counted from the immunofluorescence staining images taken from miPSCs differentiated for 14 days on TCP, GEL, or CFL conditions. (**B**) The average percentage area of DAPI (nuclei spread) was analyzed from the immunofluorescence staining images taken from miPSCs differentiated for 14 days on TCP, GEL, or CFL conditions, after no (Control) or daily treatment with the control vehicle (0.1% DMSO), ILK-specific inhibitor, Cpd22 at 0.4 μM, PAK-1-specific inhibitor, IPA3 at 1 μM, or FAK-specific inhibitor, PF-573228 at 1 μM. Comparison of means between groups. No significant differences found after Mann-Whitney U test (n=7).
